# In Vivo Confocal Microscopy in the Surgical Treatment of Keratinocyte Carcinomas: A Systematic Review

**DOI:** 10.3390/jcm14165779

**Published:** 2025-08-15

**Authors:** Monika Wojarska, Klaudia Kokot, Paulina Bernecka, Natalia Domańska, Agata Libik, Dana Bunevich, Dominika Nowakowska, Magdalena Dzido, Wiktoria Borzyszkowska, Wojciech Kazimierczak, Jerzy Jankau

**Affiliations:** 1 Plastic Surgery Department, University Clinical Center, Smoluchowskiego, 80-214 Gdańsk, Poland; jerzy.jankau@gumed.edu.pl; 2 Faculty of Medicine, Medical University of Gdańsk, M. Skłodowskiej-Curie 3a, 80-210 Gdańsk, Poland; klaudia.kokot@gumed.edu.pl (K.K.); pauline.bernecka@gumed.edu.pl (P.B.); natalia.domanska0527@gmail.com (N.D.); agata.libik@gumed.edu.pl (A.L.); dana.bunevich@gumed.edu.pl (D.B.); dominika.nowakowska@gumed.edu.pl (D.N.); magda.dzido@gumed.edu.pl (M.D.); wborzyszkowska@gumed.edu.pl (W.B.); wjkazimierczak@gumed.edu.pl (W.K.)

**Keywords:** confocal microscopy, RCM, nonmelanoma, keratinocyte carcinomas, surgical removal

## Abstract

**Background**: Keratinocyte carcinomas (KCs), including basal cell carcinomas (BCCs) and squamous cell carcinomas (SCCs), are the most prevalent malignancies globally, particularly affecting sun-exposed facial areas. Achieving clear surgical margins in these regions is essential to ensure oncologic control while preserving cosmetic outcomes. Reflectance confocal microscopy (RCM) is a noninvasive imaging technique that enables real-time, high-resolution visualization of skin structures and may aid in margin assessment during KC surgery. This systematic review aims to evaluate the role of in vivo RCM in the surgical treatment of KCs. **Methods**: This review followed PRISMA guidelines. A comprehensive search of PubMed, Scopus, Web of Science, Medline, and EBSCO databases was conducted for studies published between January 1992 and December 2024. Inclusion criteria focused on clinical studies utilizing in vivo RCM for diagnostic or surgical applications in KC management. Results: Eighteen studies involving 1112 patients were included. RCM was used preoperatively in 5 studies and intraoperatively in another 5. Nine studies assessed margin delineation, while eight focused on diagnostic accuracy. RCM improved diagnostic confidence and allowed for more precise margin assessment, potentially reducing the extent of surgical excision in cosmetically sensitive areas. However, its broader clinical adoption is limited by operator dependency, procedural complexity, and lack of standardization. **Conclusions**: RCM shows promise as a supportive tool in KC surgery, particularly for preoperative planning. While its diagnostic utility is well established, its intraoperative role requires further validation. Larger, standardized, and cost-effective studies are needed to confirm its impact on surgical outcomes and patient quality of life.

## 1. Introduction

Keratinocyte carcinomas (KCs) are the most diagnosed human cancers globally [1,2]. The term “keratinocyte carcinomas” mainly refers to basal cell carcinomas (BCC) and squamous cell carcinomas (SCC), but may also include cutaneous lymphomas, adnexal tumors, Merkel cell carcinomas, and other rare skin neoplasms [1]. BCC is the most common (75–80%), followed by SCC (15–20%) [3], with BCC being significantly more prevalent [3,4,5]. KCs arise from epidermal cells, and UV radiation exposure promotes malignant cell proliferation [6]. Thus, they typically appear on sun-exposed areas such as the neck or limbs. Lesions in the head and neck, especially in the H zone, are considered high-risk due to the likelihood of deeper invasion and recurrence [7]. This zone includes the nose, eyelids, periorbital area, ears, and central, marked by proximity to critical structures and varied skin thickness [7].

Surgical excision with a margin of healthy tissue remains the standard treatment for high-risk KCs [8]. However, unpredictable tumor growth patterns and partial spontaneous healing make it difficult to define lesion borders, increasing the risk of incomplete excision or unnecessary tissue removal—impacting oncological safety and aesthetics. While not highly lethal, KCs cause significant morbidity [9]. Based on retrospectively collected data, including a cohort of 500 patients from our center, the average excised tumor size was 14.77 mm, ranging from 2 mm to 80 mm. Even with a 2–4 mm excision margin in a histopathology exam, removing a 14.77 mm tumor can lead to irreversible damage to the eyelids, mouth, or nose, requiring complex reconstruction to restore function and quality of life.

Mohs micrographic surgery is the gold standard [4], involving layer-by-layer excision and intraoperative microscopic analysis. Only affected margins are extended, ensuring complete tumor removal with minimal healthy tissue loss [4]. Despite its precision, Mohs is costly, time-consuming, and resource-intensive, limiting patient access and increasing wait times. Early intervention is crucial, as larger lesions are harder to remove with satisfactory functional and cosmetic results.

Confocal microscopy (CM) offers a promising alternative for preoperative tumor margin assessment [10,11,12]. This laser-based imaging tool provides black-and-white images resembling histology. CM includes reflectance CM (RCM) and ex vivo CM (EVCM). RCM enables in vivo imaging with histology-like sensitivity, using only reflectance contrast, without dyes [13]. CM bridges dermoscopy and histopathology, expanding diagnostic capabilities [14,15,16,17] and enabling noninvasive assessment comparable to biopsy [18,19]. It shortens diagnostic and treatment pathways [14,15], facilitating diagnosis without tissue disruption [20]. Given these advantages, CM is also used in the surgical management of skin cancers, potentially improving cosmetic outcomes and reducing resource use. It may minimize surgical trauma, enhance recovery, and improve patients’ quality of life.

The aim of our work is to conduct a systematic review of the literature on the use of confocal microscopy (CM) in treating keratinocyte carcinomas (KCs). Despite its promise, CM’s clinical application in KC management remains inconsistent. Although several studies have evaluated its diagnostic and perioperative value, unresolved questions include standardization, reproducibility, operator dependency, efficacy across subtypes and anatomical sites, and cost-effectiveness relative to Mohs surgery. Furthermore, high-quality, large-scale studies on recurrence, surgical margins, and cosmetic and functional outcomes are lacking. Therefore, this review aims to evaluate current evidence, identify knowledge gaps, and suggest future clinical research priorities.

## 2. Materials and Methods

This study adhered to the Preferred Reporting Items for Systematic Reviews and Meta-Analysis (PRIMSA) guidelines. A systematic search of the PubMed, Web of Science, Scopus, Medline, and EBSCO databases for articles published between January 1992 and December 2024 was performed using search terms designed to identify studies on the use of CM as an adjunct method in the surgical treatment of KCs. The following search query was used: “(confocal microscopy OR confocal imaging OR RCM) AND (nonmelanoma OR bcc OR skin cancer OR scc OR keratinocyte carcinomas)” AND (surgical removal OR surgery OR Mohs OR presurgical OR intraoperative).

**Figure 1:**
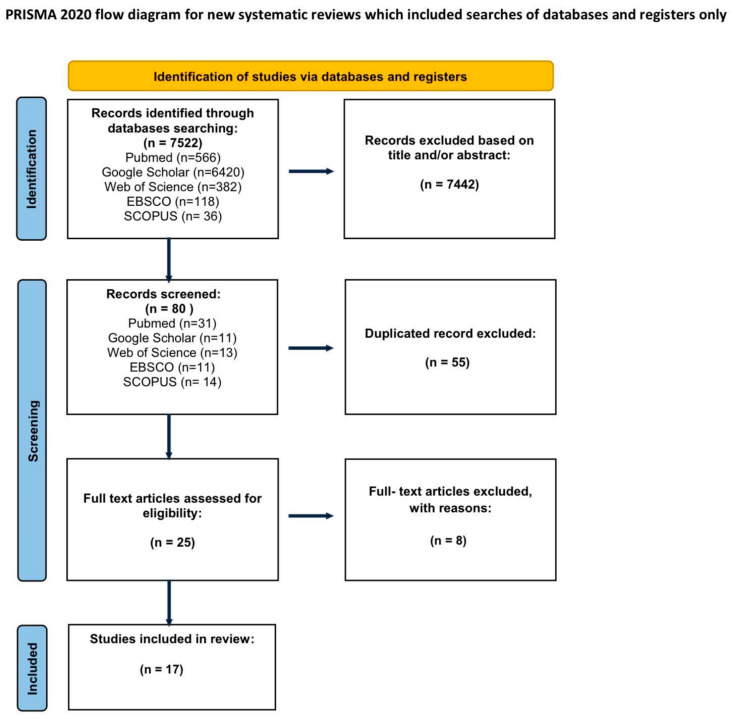
PRISMA flowchart.

Inclusion criteria were that the articles provided the use of CM as an adjunct method in the surgical treatment of keratinocyte carcinomas. The exclusion criteria involved studies that included the application of ex vivo CM, the exclusive use of CM as a diagnostic method, the use CM without marking the tumor margins, the use of CM as an adjunct method in the surgical treatment of melanoma, the application of combined techniques, e.g., optical coherence tomography (OCT), and marking the margins for removal by means other than surgery, e.g., radiotherapy or laser ablation. Unrelated articles that were discovered by keyword matching were also excluded. Abstracts, case reports, conference papers, letters, editorials, and articles written in languages other than English were excluded during the initial screening of titles and citations. Studies duplicated in databases were removed using Mendeley Software (Reference Manager Version 2.130.2). After the initial search was performed by one researcher, duplicate records were removed. Two independent reviewers screened the remaining titles and abstracts according to predefined inclusion and exclusion criteria. Based on this screening, potentially relevant full-text articles were identified and retrieved. Full-text articles were then independently assessed by the same two reviewers to determine the in vivo application of CM in the surgical treatment of skin cancer on the face. We specifically evaluated whether the study involved tumor resection, the use of RCM (in vivo and/or ex vivo), and whether clinical outcomes and conclusions relevant to CM application were reported. Disagreements at any stage were resolved by consensus in consultation with a third and fourth reviewer. Final decisions were made under the supervision of the first author. A flow diagram of the study selection process is presented in Figure 1.

The initial search identified 7522 articles. The initial search identified 7522 articles. After excluding articles that do not match the criteria, 90 studies were included for abstract review. Finally, 25 were selected for full text appraisal, of which 17 met all the inclusion criteria and were included in this review. This systematic review included 17 articles, including over 1112 patients (Table 1). Due to significant variations in the technique and purpose of RCM application, the publications are too diverse to adhere to a meta-analysis. The articles included in the review are presented in Table 1.

### 2.1. Type of Cancer

In 12 studies, the treatment of basal cell carcinoma of the skin was examined, while in 5 studies, both basal cell and squamous cell carcinoma treatments were examined.

### 2.2. Type of Surgery

In 10 studies describing 208 patients, CM was used as an adjunct method for Mohs surgery. In 7 articles, describing 904 patients, the use of RCM as an adjunct method during conventional surgical removal of skin cancer was evaluated.

### 2.3. The Use of CM

In 5 studies, excision margins were evaluated preoperatively, while in 5 studies, the margins were determined intraoperatively.

In 8 studies, the possibility of using CM as a method of confirming cancer diagnosis was evaluated, including in 4 studies concerning the use of RCM in conventional surgery on 817 patients and in 4 articles concerning its use in Mohs surgery on 158.

In 8 articles, the use of RCM to assess margins was evaluated, including in 4 studies concerning conventional surgery on 47 patients and 6 concerning Mohs surgery on 70 patients.

### 2.4. Conclusions Reported by Original Studies

Across the reviewed literature, most studies concluded that CM, and particularly reflectance confocal microscopy (RCM), holds promise as an adjunct tool in both diagnosis and surgical planning for skin cancer. Its ability to provide high-resolution, real-time imaging of the superficial dermis and epidermis was frequently cited as a significant advantage. Studies noted that CM contributed to improved diagnostic confidence and more accurate delineation of tumor margins, which in turn supported tissue-sparing surgical strategies. This was especially relevant in the management of recurrent tumors or lesions with poorly defined clinical borders.

However, several limitations were also consistently reported. These included the steep learning curve associated with image acquisition and interpretation, the time-intensive nature of the procedure, and limited imaging depth, which restricts the visualization of deeper or infiltrative tumor components. Such challenges have likely contributed to the variability in clinical adoption observed across studies.

In summary, while the extracted data confirm that CM demonstrates valuable diagnostic and intraoperative applications, its routine integration into surgical workflows remains inconsistent. This variability is largely attributable to technical constraints and the need for further standardization. The implications of these findings are discussed in greater detail in the following section.

## 3. Discussion

This systematic review provides a structured overview of currently available evidence on the use of confocal microscopy (CM), particularly reflectance confocal microscopy (RCM), in the surgical treatment of KC. While the number of included studies is limited, and data are heterogeneous, our findings highlight patterns of clinical application and point toward areas requiring further investigation [16,18,22]. Our analysis aimed to evaluate the clinical utility of CM as a complementary technique in determining surgical margins and confirming diagnosis and to assess whether it could improve the accuracy and outcomes of skin cancer surgery [29]. Given the growing interest in CM and its potential role in dermatologic surgery, this review contributes to consolidating the current understanding and identifying key knowledge gaps [13,15,17]. Although the topic remains under-researched, our findings provide an updated overview of the clinical contexts in which CM has been implemented and evaluated [12,16].

There are increasing reports on the use of CM in conventional excision and Mohs micrographic surgery [18,21,24,28,32,33]. Most studies conclude that it can improve intraoperative decision-making, especially in poorly defined or recurrent tumors [13,31]. The studies reviewed consistently indicate that CM can assist the surgeon in complete tumor resection and reduce the risk of residual disease [17,18,22,27]. Its ability to image the epidermis and superficial dermis in high resolution in vivo allows visualization of tumor architecture in real time without the need for tissue processing [12,14,15]. However, the total number of cases described in the literature remains limited—only 137 patients have been evaluated for CM-based margin assessment—highlighting that the current evidence base is narrow and comes from small, heterogeneous patient cohorts [16,23,30].

Several limitations probably contribute to the fact that CM is rarely used in the assessment of surgical margins. First, the method is very time-consuming and requires expert interpretation and manual scanning of lesion margins [13,23,34]. Second, the variability of image acquisition protocols and the lack of standardized criteria for defining the positivity of margins limit reproducibility and clinical acceptability [20,26,29]. In addition, CM is generally limited in its ability to visualize deeper dermal structures, which may be a disadvantage in infiltrating or deeply invasive subtypes of BCC [18,22,31]. These factors may partly explain the muted clinical enthusiasm for CM in the assessment of margins, despite its theoretical advantages [13,15].

In contrast, the use of CM for diagnostic purposes is supported by more robust data. In 995 patients included in the studies examined, CM showed high sensitivity and specificity in identifying lesions, with performance metrics comparable to those of histopathology [19,20,29,30]. These results suggest that CM could play a more important role in non-invasive diagnosis or as a triage tool prior to surgical planning [13,14]. However, some authors reported that CM was less effective when used intraoperatively in the context of Mohs surgery, possibly due to technical limitations such as difficulties in correlating confocal images with frozen section histology or delays in acquisition and interpretation [27,28,33].

Despite these limitations, CM is particularly promising in selected patient groups and clinical scenarios. In young patients or when treating lesions in cosmetic or functional risk areas such as the central face, eyelids, or lips, precise margin control is crucial to avoid overtreatment and reduce the need for extensive reconstruction [12,17,25]. In such cases, CM can support a customized surgical approach that limits excision to the affected areas while preserving healthy tissue [28]. In addition, the non-invasive nature of CM makes it particularly attractive for situations where biopsy is contraindicated or undesirable [14,29,35].

Our review also demonstrates that conclusions reported by the included studies were generally aligned in recognizing CM’s potential clinical value. Across the articles, CM was frequently associated with improved visualization of tumor architecture, more informed intraoperative decisions, and enhanced diagnostic accuracy [13,16,22]. At the same time, multiple studies noted the technical and procedural barriers to its wider adoption, including operator dependency, cost, and limited availability [20,23,29].

The current literature is limited not only by small sample sizes but also by methodological inconsistencies and insufficient data on long-term outcomes. A key limitation of the current evidence is the lack of stratification by tumor subtype and histological differentiation. Most studies did not distinguish between low-risk and high-risk BCCs, nor did they report SCC differentiation grades consistently [3,5,19,29]. This is clinically relevant, as more aggressive tumors may extend beyond the imaging capabilities of confocal microscopy [16,31,34]. Without subgroup analysis, it is difficult to assess CM’s effectiveness in complex cases where precise margin control is essential. Future studies should address this gap by explicitly classifying tumors to better evaluate CM’s diagnostic and intraoperative value, as well as aim to validate the performance of CM in larger, multicenter cohorts using standardized imaging protocols and objective endpoints [13,16,23,30]. In addition, cost-effectiveness analyses are needed to determine whether the integration of CM into routine clinical practice offers measurable benefits in terms of lower recurrence rates, lower costs to the healthcare system, or improved patient-reported outcomes [29].

In summary, confocal microscopy appears to be a promising adjunct in the surgical treatment of KCs, especially when used in well-selected cases [13,16,18,22]. The findings from this review support the role of CM primarily in diagnostic settings and, to a more limited extent, in intraoperative margin evaluation [19,20,27]. Broader implementation will require technological improvements, procedural standardization, and stronger clinical evidence [14,23,30]. Standardization of CM protocols and further exploration of its clinical value are needed to fully define its role in dermatologic surgery [16]. Given the increasing incidence of skin cancers worldwide, any tool that can improve surgical precision and minimize the risk of recurrence deserves close attention and further investigation [1,2,8].

## Figures and Tables

**Table 1:** Articles included in the systematic review.

Authors	Type of Surgery	Number of Patients	Type of Use RCM	Type of Cancer	The Effect of Using RCM	Timing of RCM Use	Confocal Microscope Type	Depth Limit (μm)
Pan et al. 2012 [21]	CLASSIC REMOVAL	9	EXCISION MARGIN	BCC	Objective: The accuracy of RCM in determining the lateral margins of BCC in vivo compared to histology was 92.3%.	PREOPERATIVELY	Vivascope 1500	200
Venturini et al. 2016 [17]	MOHS	3	EXCISION MARGIN	BCC	Subjective: Preoperative results corresponded to the results of intraoperative histopathological examination.	PREOPERATIVELY	Vivascope 1500	200
Lupu et al. 2021 [22]	CLASSIC REMOVAL	7	EXCISION MARGIN	BCC	Objective: The accuracy of RCM in determining the lateral boundaries of BCC in vivo compared to histology was 93.1% (95% CI 0.77–0.99), with a sensitivity of 66.67% and a specificity of 100%.	PREOPERATIVELY	VivaScope	200–250
Richarz et al. 2022 [23]	MOHS	17	EXCISION MARGIN	BCC	Objective: No significant differences between the RCM-predicted and the actual surface area of the surgical defect, 2.95 cm^2^ vs 2.52 cm^2^.	PREOPERATIVELY	Vivascope 3000	200–250
Tannous et al. 2003 [24]	MOHS	5	EXCISION MARGIN	BCC	Subjective: Confocal microscopy in vivo can be a useful tool in supporting Mohs surgery, and aluminum chloride effectively enhances tumor contrast in this imaging technique.	INTRAOPERATIVELY	Vivascope 1000	No information
Gualdi et al. 2016 [25]	CLASSIC REMOVAL	1	EXCISION MARGIN	BCC	Objective: RCM detected that islands of BCC cancer extended 0.1 mm beyond the initial incision. Subjective: Allowing the surgeon to redraw the surgical margin (implying a positive impact).	INTRAOPERATIVELY	No information	No information
Teixeira et al. 2018 [26]	MOHS	8	EXCISION MARGIN	BCC	Objective: In six cases (50%), the margins were free of cancer in the first stage of MMS. Subjective: RCM enabled full determination of the lateral boundaries of the tumor in all cases, leading to the preservation of healthy skin.	INTRAOPERATIVELY	Vivascope 1500	300
Flores et al. 2019 [27]	MOHS	17	EXCISION MARGIN	BCC, SCC	Objective: RCM videos and video-mosaics showed imaging quality in 91% of NMSC lesions pre-operatively and 83% intra-operatively. Sensitivity/specificity were 71%/86% and 86%/81% for two RCM video-mosaic evaluators, and overall agreement was 80% and 83% with histopathology. Subjective: The imaging quality was acceptable (resolution and contrast)	PREOPERATIVELY, INTRAOPERATIVELY	Vivascope 3000	120–150
Shavlokhova et al. 2021 [28]	CLASSIC REMOVAL	50	EXCISION MARGIN	BCC	Objective: RCM in vivo identified BCC at wound margins with a sensitivity of 88.5% and a specificity of 91.7% compared to imaging of intact skin, and with a sensitivity of 97.8% and a specificity of 90.7% compared to histopathology. Subjective: This method allows for the identification of BCC at wound margins.	INTRAOPERATIVELY	Vivascope 3000	350
Ahlgrimm-Siess et al. 2018 [29]	CLASSIC REMOVAL	224	DIAGNOSTIC METHOD	BCC	Objective: Sensitivity of 97% and specificity of 93% were demonstrated in the diagnosis of BCC	PREOPERATIVELY	Vivascope 1500	250
Navarrete-Dechent et al. 2019 [10]	MOHS	47	DIAGNOSTIC METHOD	BCC	Objective: Sensitivity, specificity, positive predictive value, and negative predictive value of RCM were 92.8%, 68.4%, 86.6%, and 81.2%, respectively. Subjective: Due to the strong correlation between RCM imaging and histopathological outcomes, RCM can be a valuable tool for assessing residual BCC at the biopsy site, which may help reduce the number of unnecessary surgeries.	PREOPERATIVELY	Vivascope 3000	200
Stefanski et al. 2024 [30]	CLASSIC REMOVAL	410	DIAGNOSTIC METHOD	BCC, SCC	Objective: 50.6% of patients avoided biopsy, and the correlation between RCM and histopathology was 82.76%. Subjective: Thanks to RCM, patients avoided biopsy (implying a positive outcome).	PREOPERATIVELY	Vivascope 1500	200
Schüle et al. 2009 [31]	MOHS	66	DIAGNOSTIC METHOD	BCC	Objective: The concordance coefficients between RCM and histopathology were low. Subjective: The effect was poor.	INTRAOPERATIVELY	Vivascope 2500	No information
Scope et al. 2010 [32]	MOHS	20	DIAGNOSTIC METHOD	BCC, SCC	Objective: Wounds were fully visualized using RCM in 33% of cases (out of *n* = 39), and in 4 cases, RCM visualized BCC, later confirmed histologically. Subjective: RCM imaging of wounds is feasible but limited in visualizing all cases.	INTRAOPERATIVELY	Vivascope 2000	300
Flores et al. 2015 [33]	MOHS	25	DIAGNOSTIC METHOD	BCC, SCC	Subjective: Due to the good correlation of RCM imaging with histopathology, RCM shows potential in intraoperative detection of cancer presence at wound edges, which could shorten surgery time and improve its effectiveness.	INTRAOPERATIVELY	Vivascope 3000	150
Shavlokhova et al. 2022 [34]	CLASSIC REMOVAL	62	DIAGNOSTIC METHOD	BCC	Objective: In seven of 10 (70%) cases, cancer margins were identified; in three of 10 (30%) cases, margins could not be detected. All frozen biopsies of surgical margins were negative, and in 12 of 13 (92.3%) cases, RCM margins were negative. Subjective: The effect was partial, and with a larger database, greater effectiveness in automatic detection of BCC is possible.	INTRAOPERATIVELY	Vivascope 3000	No information
Ferrari et al. 2017 [35]	CLASSIC REMOVAL	141	DIAGNOSTIC METHOD	BCC, SCC	Subjective: RCM is a valuable diagnostic tool that allows for in vivo imaging of tissues, contributing to more accurate diagnostics of NSC of the head and neck, saving patient time and costs for the public healthcare system.	PREOPERATIVELY	No information	No information

## Data Availability

Available on request.
